# Characterization and Making Techniques of Calcareous Construction Materials for Phaya Thon Zu Temple in Bagan Historical Area, Myanmar

**DOI:** 10.3390/ma17174294

**Published:** 2024-08-30

**Authors:** Hye Ri Yang, Gyu Hye Lee, Dong Min Kim, Chan Hee Lee

**Affiliations:** 1 Department of Cultural Heritage Conservation Sciences, Kongju National University, Gongju 32588, Republic of Korea; 1212heri@naver.com (H.R.Y.);; 2 Conservation Science Division, National Research Institute of Cultural Heritage, Daejeon 34122, Republic of Korea; 3 International Cooperation Center, Korea Heritage Agency, Seoul 06153, Republic of Korea

**Keywords:** Phaya Thon Zu temple, mortar, plaster, stucco, manufacturing techniques

## Abstract

The calcareous materials used in constructing the Phaya Thon Zu temple at the Bagan historical sites in Myanmar are mortars, plasters, and stuccos. Among them, the mortars and plasters are a mixture of original and new materials used for recent conservation treatments. In this study, the making techniques were examined through analysis of calcareous materials by production period. All calcareous materials have a mineral composition similar to soil, except calcite. Stuccos have the most refined aggregates, homogeneous particle size, and the highest lime and organic contents. They were designed to improve ease of carving and weathering resistance, considering the unique characteristics of the stuccos. Because all calcareous materials were mixed with soil, the origin of the clay materials was analyzed. It was concluded that the mortars were produced by mixing clay and sandy soil, and the original mortars showed characteristics similar to soil. It is highly possible that sandy soil from around the Htillominlo temple was used to produce new plasters, and it is estimated that a mixture of clay soil was used for the original plasters and stuccos. A clear provenance interpretation of the original and raw materials used for each construction and the mixing ratio of clay materials need to be discussed through experiments, along with the estimated provenance area of the raw calcareous materials.

## 1. Introduction

**Figure 1:**
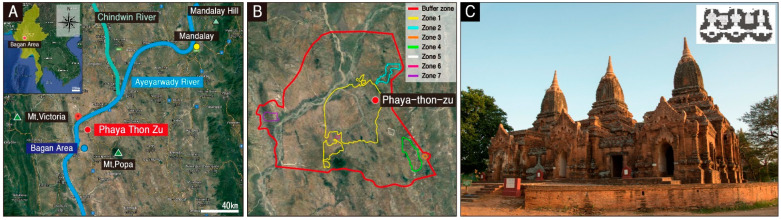
Location and overview of the study area. (**A**) Detailed location of the Phaya Thon Zu temple and Bagan area in Myanmar. (**B**) Map showing the Bagan historical area. (**C**) General view of the Phaya Thon Zu temple.

The Bagan historical area is located at a bend in the Ayeyarwady River developed in the central plains of Myanmar, and it is one of the three greatest Buddhist sites in the world designated by UNESCO, along with Angkor monuments in Cambodia and Borobudur temple in Indonesia, with the most enormous scale among them (Figure 1A). Numerous archaeological sites, such as pagodas, temples, and monasteries, as well as murals and sculptures, are distributed in the Bagan region [1]. They are a testament to the climax of the Bagan civilization in Myanmar, which flourished during the 11th to 13th century, and its dedication to Buddhism [2]. In recognition of their value, seven sectors of Bagan were registered as UNESCO World Heritage Sites in June 2019 (Figure 1B).

The subject of this study, the Phaya Thon Zu temple, with the literal meaning of three pagodas, is believed to have been constructed in the late 13th century [1,3]. It consists of three independent temples with antechambers and a main chamber covered with domed ceilings that are connected by two passages, a unique structure that differentiates it from other temples in the Bagan area (Figure 1C). The antechamber of each temple houses a primary Buddha statue, and the interior walls and ceilings are decorated with a wide range of artistic elements, including various murals such as painted representations of Sakyamuni Buddha statues and lotus patterns, thereby endowing a significant archaeological and historical value [4].

It is believed that approximately 4000 pagodas were constructed between the 11th and 13th centuries in the Bagan area, and there are currently about 3000 pagodas and temples distributed throughout the region. However, although many of these pagodas and temples were damaged by earthquakes with magnitudes of more than six on the Richter scale that occurred in 1975 and 2016, there is insignificant research on their repair and conservation management [5,6,7,8,9,10].

Diversified damages to the Phaya Thon Zu temple have also progressed significantly due to damage from prolonged exposure to natural weathering, disasters, and artificial deteriorations [11]. Due to the absence of technology to cope with frequently earthquakes and anthropogenic damages, it is essential to conduct scientific analysis and precision diagnosis to research on the selection and application of alternative materials that reflect the results thereof [6,12].

Therefore, based on the investigation history and records of local experts, the original and new materials used for repair works were distinguished, and various specimens of the original and new ones were obtained from multiple repair processes on the site. In this study, the same analysis method was applied to all acquired specimens to examine the differences and homogeneity between the new ones for repair work and the original materials.

Investigations on the main calcareous materials of architectural and cultural heritages have also been conducted relatively diversely in Korea [13,14,15,16,17,18,19]. However, research on the calcareous materials used as the functional materials of the primary structures has been only limitedly reviewed, thereby needing substantially more extensive studies in the future [6,7,20,21].

This study strove to analyze the material characteristics of mortars, plasters, and stuccos used in the construction of the Phaya Thon Zu temple and examine the construction technology system based on the results obtained. Accordingly, the data gathered in this study contributes towards the establishment of appropriate conservation measures to maintain the outstanding universal value, authenticity, and integrity of the original form of the damaged construction materials as a world heritage.

## 2. Materials and Methods

### 2.1. Conservation and Classification of Materials

**Figure 2:**
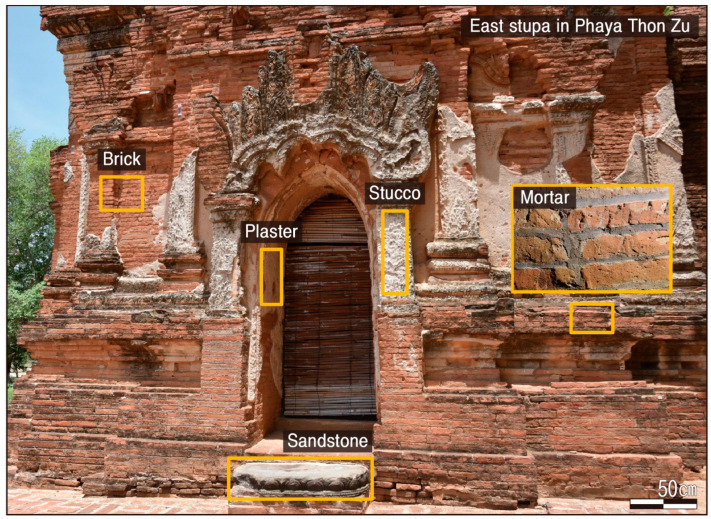
Photographs showing representative construction materials of the Phaya Thon Zu temple.

Materials including brick, mortar, plaster, stucco, and rock were utilized in the construction of the Phaya Thon Zu temple (Figure 2). Bricks were made by firing clay and were the structural material used in the construction of the entire temple. Almost all have a reddish-brown color, are rectangular in shape, and are classified into small and large bricks with relatively constant dimensions of about 18 × 10 × 5 cm and 28 × 16 × 3 cm, respectively.

Mortar was used as the joint filler of the bricks, which is categorized into two types: red series, for which the clay is the main component, and light gray series, for which the lime is the main component. In addition, the surface is composed of brick and mortar and finished with plaster. Plaster is made up of lime and sand as the main components and was used as the finishing material for the base layer of the murals, walls, and ceilings inside the temple at the time of construction. It is also the material used most often to repair temple walls nowadays.

Although the Phaya Thon Zu temple is situated on relatively firm ground and maintains a stable structure, a closer examination reveals that damages are distinct and progressive for each type of material. Moreover, due to the repeated repairs and numerous conservation treatments, the distinction between the original materials and the new materials used for repairs is ambiguous, and it is difficult to identify trends or uniformity in the locations at which conservation treatments were rendered.

**Figure 3:**
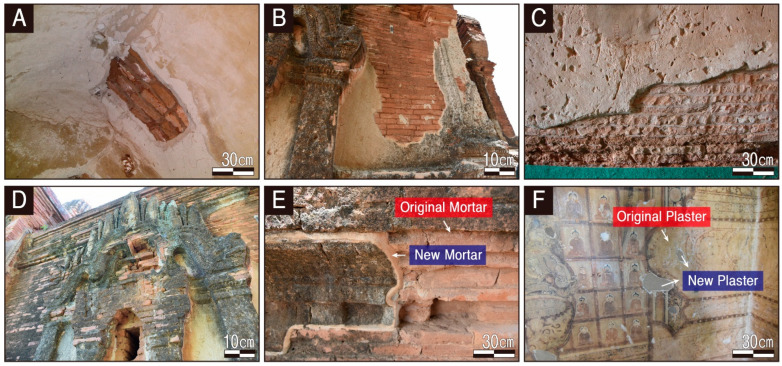
Photographs showing representative deteriorations and conservation treatments of the Phaya Thon Zu temple. (**A**,**B**) Physical damage. (**C**,**D**) Chemical and biological damage. (**E**,**F**) Examples of conservation and restoration interventions.

The representative types of damages in the interior and exterior of the temple can be broadly categorized into physical damages, such as cracks, exfoliation, and detachment, and chemical and biological damages, such as discoloration by contaminants and infestation. Physical damages are particularly evident in a wide area with cracks and detachment of the brick, mortar, and plaster layers that make up the walls (Figure 3A).

Although it is presumed that the stuccos covered the entire exterior walls of the temple at the time of construction, much of it has fallen and is lost due to natural deterioration and natural disasters, such as earthquakes. The physical damages in the remaining stuccos are also very severe, with an extensive distribution of cracks and detachment throughout the entire area (Figure 3B).

Chemical and biological damages are also observed throughout the temple. The temple’s interior and exterior brickwork and calcareous auxiliary materials are interspersed with cavities throughout. These appear to have been caused by insects, implying the possibility of the addition of organic components, such as molasses or sap, at the time of brick production (Figure 3C). Among the calcareous materials, the stuccos used for exterior decoration displayed the most pronounced biological contamination with extremely severe black stains and lichen coverage, making it impossible to discern its original color (Figure 3D).

Although the conservation history of the Phaya Thon Zu temples has still not been documented or confirmed, it is presumed that conservation measures were taken intermittently, over several periods, based on the lack of consistency of locations and methods of repair implemented. It can be confirmed that the original mortar used as joint filler has been overlaid with relatively recent new mortars along the sections of cracks and detachments (Figure 3E). The sections with detached coloring layers of the mural are interspersed with traces of relatively recent repairs with new plasters (Figure 3F).

The original materials presented in the study and the new materials used for the repair are distinguished visually on site. In particular, the two parts differ in color and texture. However, in order to obtain samples for analysis, the original and new repair mortars were selected with the help of local experts who were in charge of the actual repair of the temple.

### 2.2. Analytical Specimens

In this study, samples of 6 mortars, 6 plasters, and 11 stuccos were used to analyze the material characteristics of the materials used at the time of the initial construction and the new materials used in repairs after that. All the stuccos are original. In the case of the samples of mortars used as joint fillers for the bricks, some are original and some are new; the first ones are brown, and the second have a milky white hue.

**Figure 4:**
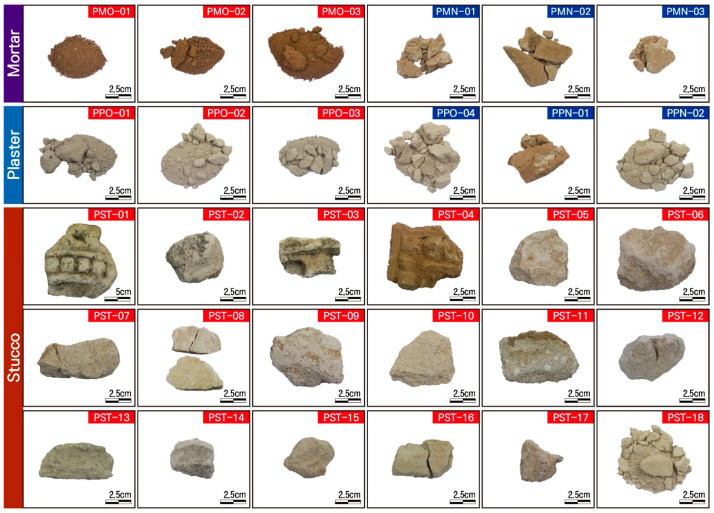
Photographs showing the samples analyzed. Sample numbers are the same as those of Table 1.

The original mortars have been detached from the temple’s exterior walls in a powdery state and have a clay-like consistency with a high alumina content. As mentioned above, the original mortar and the mortar used for repair have differences in color and texture. On the other hand, the new mortars are relatively cohesive and have a distinct calcareous substrate (Figure 4, Table 1).

The sample selection for the study was quite limited. However, since the temple had already undergone several repairs, there were relatively many and diverse samples preserved on site. Fortunately, through discussions with the local administrator, we were able to secure the minimum number of samples required for analysis.

Two of the plaster samples are new materials used for the repairs. The original plaster was collected from those detached from the exterior wall in a powdery state, along with the agglutinated portion with a milky white color. The new plaster, in part, has a relatively high degree of solidification, a grayish-brown color, and a mixture of white substances, thereby displaying different characteristics.

Also, all the stucco samples are in their original form, produced at the time of the initial construction. Although they generally have a milky white color, some samples have a light brown color. Even though they display decorative features that may have adorned the temple’s exterior walls, their decorative elements are either unclear or unconfirmed since some samples were collected only in minute quantity. The stucco samples are generally dominated by calcareous substrate, but phaneritic crystals were not confirmed (Figure 4).

### 2.3. Research Methods

To examine the material characteristics of the calcareous auxiliary materials used in the Phaya Thon Zu temple, a precision field survey of the construction materials that compose the temple was conducted first to collect calcareous samples that fell off by the time of their respective productions. Their physicochemical characteristics, such as chromaticity, magnetic susceptibility, and P-XRF analysis, were reviewed on the site to select the representative samples based on their outcomes.

The studied samples were concurrently examined under a stereomicroscope (Nikon Eclipse LV 100N POL, Nikon Instruments Inc., Melville, NY, USA) to investigate the status of their yield, voids, mineral composition, and weathering. Thin sections were also prepared and examined under a polarizing microscope (Nikon Eclipses E 600W, Nikon Instruments Inc., Melville, NY, USA), and scanning electron microscopy (Tescan, MIRA-3, Brno, Czech Republic) and attached energy-dispersive elemental spectroscopy (Bruker Quantax 200, Bruker, Bremen, Germany) were used for microstructure and qualitative analysis. The samples were coated with platinum (Pt) to increase their electrical conductivity and minimize the influence of the composition ratio.

To precisely identify the mineral composition of all samples, they were pulverized into sizes less than 20 μm and subjected to X-ray diffraction analysis (Rigaku MiniFlex 600, Rigaku, Tokyo, Japan). The X-ray irradiation source was CuKα, and the anode accelerating voltage and filament current were 15 mA at 40 kV, respectively. The measurement range and speed were 3° to 60° and 1°/min, respectively.

Meanwhile, a differential thermal analyzer and thermogravimetric analyzer (TA Instruments, SDTQ600, Hüllhorst, Germany) were used to track the thermal history and phase transitions of the constituent minerals during the reheating process of the representative samples. The α-Al_2_O_3_ was used as a standard sample, and the thermal history change was measured in the room temperature range to 1000 °C at a rate of 10 °C/min.

Geochemical analysis was performed to quantify each sample’s major, some trace, and rare earth elements. Analyses were performed at ACT LAB in Canada using inductively coupled plasma-atomic emission spectroscopy (ICP-AES), mass spectrometry (ICP-MS), and an instrumental neutron activation analyzer (INAA). In addition, all analytical data were validated for their quantitation by utilizing blank, duplicate, and standard samples.

## 3. Results and Interpretation

### 3.1. Textural Characteristics

**Figure 5:**
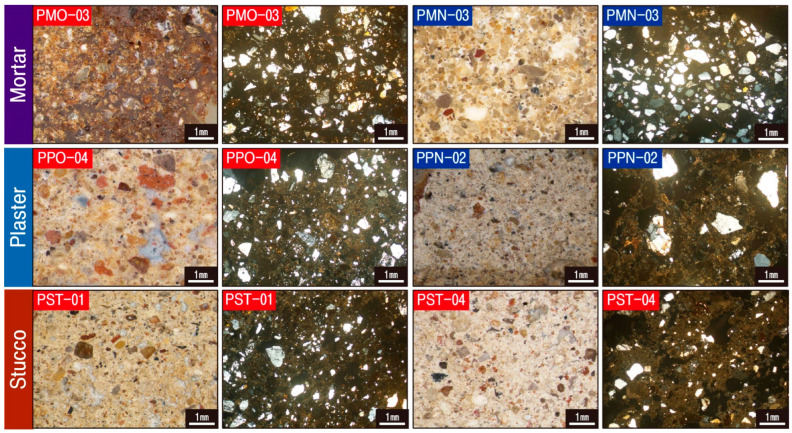
Photographs showing representative stereoscopic and polarizing microscopic images of calcareous materials. Sample numbers are the same as those of Table 1.

**Figure 6:**
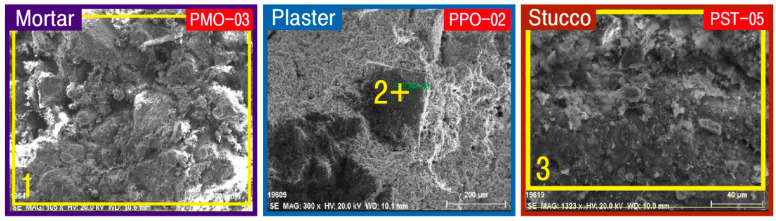
Microphotographs showing the SEM images and analytical area for SEM-EDS of calcareous materials. Sample numbers are the same as those of Table 2.

To examine the microtexture of the calcareous materials to be investigated, including the substrate, particle size of minerals, voids, and organic matter distribution, stereoscopic and polarizing microscopy observations and SEM-EDS analysis were conducted. As a result, although the substrate of all the mortars utilized as joint sealers was cryptocrystalline regardless of their production time, a relatively large quantity of clay was observed in the substrates of the raw materials (Figure 5). The constituent minerals are mainly quartz, with a size of approximately 1 mm and a roundness ranging from subcircular to subangular. Such characteristics were also visible under SEM, and EDS analysis of some raw materials displayed the highest content of Si (Figure 6, Table 2).

Plaster appeared as a covering layer on bricks and mortar, forming the base layer of murals. According to the microtextural characteristics of the different production periods, the original plaster was a yellowish cryptocrystalline matrix with the detection of many subangular quartz and opaque minerals with sizes of about 1 mm. The new application had a milky white microcrystalline substrate with observation of subangular to angular quartz with dimensions of more than 1 mm, thereby illustrating some differences in composition minerals and grain sizes depending on the production time (Figure 5). Under the scanning electron microscopy, although massive substrate was dominant in both the original and new plasters, calcite of idiomorph crystals was observed in the original plaster (Figure 6, Table 2).

The stucco displayed milky white to the white microcrystalline substrate without significant differences, although some samples (PST-1, PST-4) had high chrominance values and others (PST-5) had low chrominance values. They generally contained a significant quantity of poorly apportioned subangular to angular prismatic quartz with sizes of around 1 mm, along with observation of a small amount of clayey material and opaque minerals (Figure 5). Numerous organic matters along with the peculiar substrate were confirmed in SEM-EDS analysis with the detection of Ca with the highest content at 20.94 wt.% and Si and Fe subsidiary component elements (Figure 6, Table 2).

### 3.2. Mineralogical Compositions

**Figure 7:**
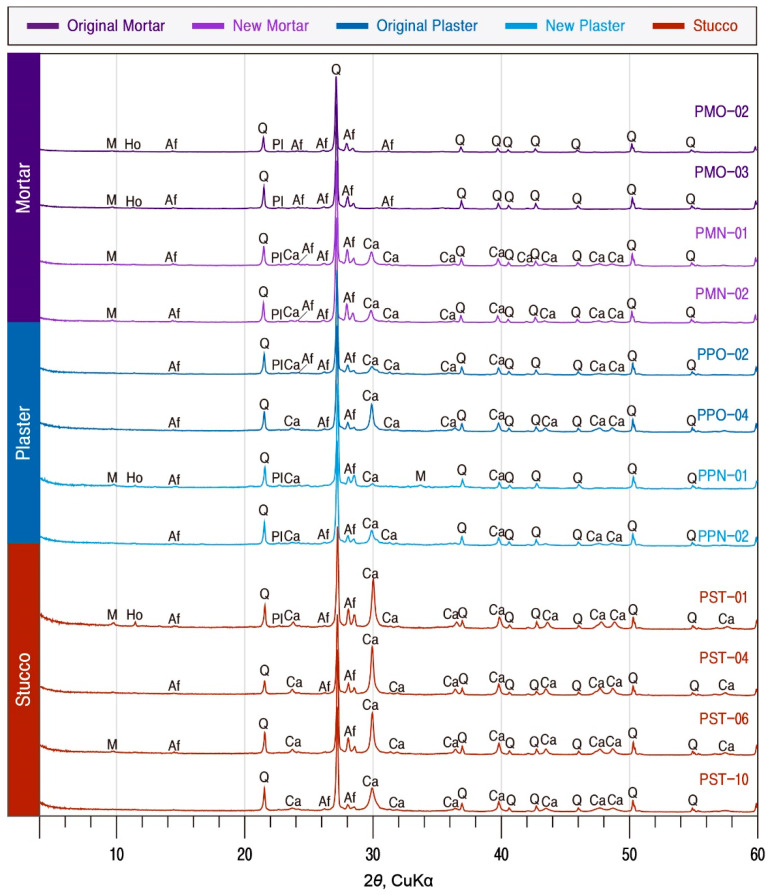
X-ray diffraction analysis of calcareous materials. M; mica, Ho; hornblende, Af; alkali feldspar, Q; quartz, Pl; plagioclase, Ca; calcite. Sample numbers are the same as those of Table 1.

X-ray diffraction analysis was performed to investigate the mineral composition of the calcareous materials. As a result, quartz, alkali feldspar, plagioclase, mica, and amphibole were detected in the original mortar. In contrast, calcite appeared in the new mortar and amphibole was not seen in the new mortar. Thus, mortars used as joint fillers displayed differences in their mineral compositions depending on the time of production and utilization (Figure 7).

Quartz was the most dominantly detected mineral in the plaster regardless of the production, and calcite and alkali feldspar are commonly confirmed as subordinate minerals. The mineral composition of some of the new plasters used in the repair displayed some variations with the presence of mica and amphibole. However, secondary minerals that can occur from the hydration reaction of calcareous materials did not appear, so more quantitative analysis was required to detect them.

Although the minerals comprising the stucco were mainly quartz, SEM-EDS analysis displayed a high content of Ca, implying that the microcrystalline substrate contains numerous minerals with Ca as the main element. As a result of X-ray diffraction analysis of the stucco, a mineral composition mainly of calcite and quartz was found with alkali feldspar, mica, and traces of amphibole (Figure 7).

### 3.3. Thermal Analyzes

The analyzed calcareous materials were composed by mixing raw materials with different composition ratios, and although their mineral composition could be determined by X-ray diffraction analysis, it was not easy to discern the changes in the mineral phase. Therefore, differential thermal analysis (DTA) and thermogravimetric analysis (TGA) were performed concurrently to track the changes in the mineral phases and weight loss due to temperature change.

**Figure 8:**
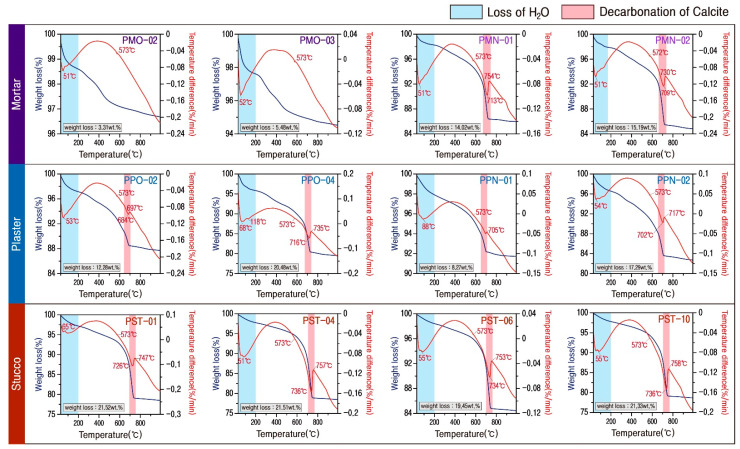
Representative DTA-TG diagrams of calcareous materials. Sample numbers are the same as those of Table 3.

The mineral composition of the mortar varied depending on the production time, and the thermal characteristics were also different. As a result of thermal analysis, the original mortar had an average weight loss of 4.40 wt.%, while the modern repair material had a very high weight loss of 14.61 wt.% (Figure 8, Table 3). In addition, only a 573 °C endothermic peak by the phase transition of quartz, along with an endothermic peak in the low temperature domain, was confirmed in the raw material.

However, an endothermic reaction at about 750 °C is confirmed simultaneously, along with a large amount of weight loss at around 750 °C in the new mortar (Figure 8, Table 3). This peak is interpreted as a characteristic of the decarbonization reaction of calcite. Therefore, proving that the analyzed mortar has different mineral compositions depending on the production time was possible.

The plaster displayed an endothermic peak at 573 °C and an endothermic response at around 700 °C due to the phase transition of quartz in all samples (Figure 8). Moreover, there was a sharp weight loss along with an endothermic reaction at around 700 °C due to the decarbonization reaction of calcite. The weight loss rate of the whole plaster was relatively high, ranging from 8.27 to 20.48 (average of 14.58) wt.%, and the reduction rate of the original plaster was high (Table 3).

Stuccos displayed the highest weight loss rate, ranging from 19.45 to 21.52 (average 20.95) wt.% (Table 3). They had a common trend of weight loss that decreases slightly up to about 680 °C and then sharp decreases after that. Along with the weight loss, an extensive range of endothermic peak at about 750 °C caused by the decarbonization reaction of calcite was detected in all samples (Figure 8).

### 3.4. Geochemical Analyzes

The geochemical characteristics of the studied calcareous samples were analyzed to quantitatively review their material characteristics and homogeneity (Table 4). Major and some trace elements of all calcareous materials were standardized based on the average compositions of rocks presented by [22,23], respectively. The initial ratio of meteorite presented by [24] was applied to rare earth elements and the primitive mantle compositions presented by [25] to compatible and incompatible elements.

**Figure 9:**
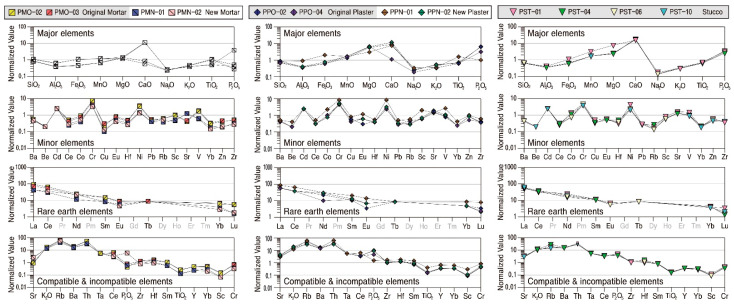
Normalized pattern diagrams showing the major, minor, rare earth, compatible and incompatible elements of calcareous materials. Sample numbers are the same as those of Table 1.

The mortar had the highest SiO_2_ content with an average of 68.84 wt.%. It contained CaO (average 7.82 wt.%) and Al_2_O_3_ (average 7.01 wt.%) as subsidiary components (Figure 9). The contents of all the elements are differentiated according to the time of their production, and, in particular, the CaO content is high in the new mortars from which calcite was detected. The original mortar with low CaO content has high Al_2_O_3_, K_2_O, Fe_2_O_3_, MnO, and TiO_2_ contents, while the MgO content was similar regardless of the time of production but varied depending on the content of SiO_2_ (Table 4). Although the compatible and incompatible elements had similar overall behavior, they differed slightly in their levels of enrichment and deficiency, while P_2_O_5_ content varied by the samples.

In particular, although the original mortar had a high alumina content with no detection of calcite, the new mortar displayed the properties of a lime mixture. Even though quartz and feldspar are also detected in the new mortar obviously, their physical and mineralogical characteristics were significantly different from those of the original mortar. However, despite the differences in material characteristics by the time of their projection, their geochemical behavior tended to be similar (Figure 9, Table 4).

SiO_2_ content was the highest, with an average of 55.31 wt.% in all plasters, followed by CaO and Al_2_O_3_, with average contents of 13.93 wt.% and 7.04 wt.%, respectively. When the correlation of elements with SiO_2_ was examined, CaO content was found to be higher, and MgO content was also relatively higher in the original plaster than in the new plaster. However, the contents of K_2_O and Na_2_O were relatively low (Figure 9). Although the contents of trace and rare earth elements also varied somewhat depending on the extent of enrichment and deficiency of the main component element, the overall trend was very similar.

The behavior of the main element of stuccos is highly homogeneous, with SiO_2_ being the highest at an average of 45.96 wt.%, followed by CaO and LOI at averages of 22.51 wt.% and 20.37 wt.%, respectively (Figure 9). However, level enrichment of CaO, MnO, MgO, and P_2_O_5_ with the most significant level of enrichment for CaO was displayed. Trace elements had a homogeneous tendency to be enriched for Cd, Cr, Ni, Sr, and V while deficient for other elements.

In particular, some rare earth elements in stuccos displayed the tendency of a gradual decrease in the level of enrichment from light to heavy rare earths, and all the compatible and incompatible elements also behaved by displaying the same enrichment and deficiency. Therefore, since the stuccos had very homogeneous geochemical characteristics, it can be interpreted that raw materials with the same cause of their genesis were utilized (Figure 9, Table 4).

## 4. Discussion

### 4.1. Material Considerations

This study examined the material scientific diversity of the calcareous materials utilized in the construction of the Phaya Thon Zu temple by categorizing them into original and new materials. In particular, plaster and stuccos are distinguished by whether they are used as decorative elements on the exterior walls of the temple or not, and, upon summarization of their material analysis, they displayed very similar textural, mineral, and geochemical characteristics.

**Figure 10:**
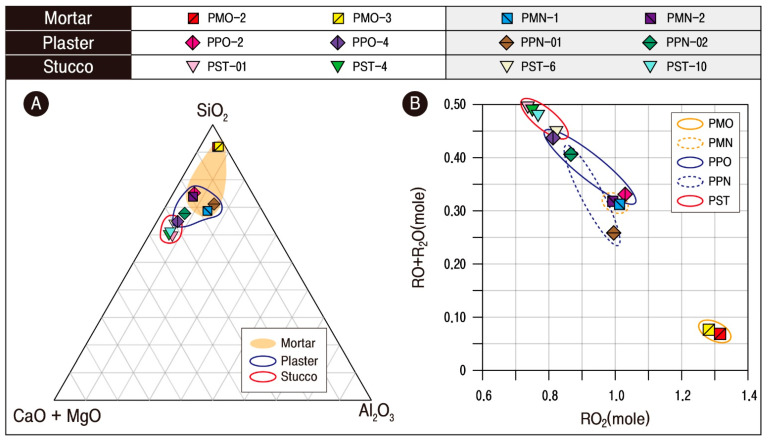
Plotted diagrams showing the comparison results of calcareous materials. (**A**) Ternary diagram showing the relationship between SiO_2_-(CaO + MgO)-Al_2_O_3_. (**B**) Correlation of (RO_2_-RO) + R_2_O. Sample numbers are the same as those of Table 4.

Therefore, to examine the differences in the characteristics of the lime mixtures based on CaO, the distribution of the main component elements was reviewed by illustrating them on the SiO_2_-(CaO + MgO)-Al_2_O_3_ triangle (Figure 10A) and preparing a basic oxide (RO + R_2_O) correlation chart by the acidic oxide (RO_2_) (Figure 10B). As a result, the contents of CaO and basic oxides were relatively the highest in stuccos.

The stuccos had a relatively good distribution compared to the plaster and mortar, with high roundness and small particle size. While the other materials displayed different appearances with low roundness and uneven distribution (Figure 5). As illustrated, mixing a higher proportion of lime in the selected aggregates with a homogeneous particle size would have been advantageous for the ease of carving and detailed expressions. It seems that the lime used to make stucco would have been more readily available based on the fact that the diversity of basic oxides acts to lower the melting point of the subject material for firing.

The function of stucco is to protect walls exposed to the external environment and prevent water leakage by sealing the joints between the individual masonry pieces and the joint filler as finishing materials. According to the archaeological evidence such as inscriptions and murals, it can be discerned that organic materials such as buffalo hide glue, Aegle Marmelos sap, fishtail palm fruit, and neem tree resin were used as additives in the production of stucco in construction works during the Bagan Dynasty [7,26,27,28].

Accordingly, given the unique nature of the stuccos being continuously exposed to the outside environment by decorating the outermost aspects of the structures, it is deemed that an increase in the binding force and enhancement of the durability were sought by increasing the contents of organic additives in their production. This may explain why the stuccos have slightly higher lime and organic component content than the finishing materials and joint filler inside the temple.

However, the effectiveness of organic additives has yet to undergo a stage of scientific verification because it is not possible to quantitatively evaluate the organic components only by means of the LOI. In addition, it would be necessary to investigate the exact composition and mixing ratio of bonding material, aggregates, and organic additives used in the lime mixture, including the finishing material and joint filler. As such, for a clear material distinction between lime mortar, plaster, and stuccos, the characteristics of organic components used as additives, the mixing ratio of raw materials, and the strength characteristics of lime mixtures must be researched.

Although the original mortars displayed characteristics close to ordinary soil, such as shallow CaO content and almost no detectable calcite, the new mortar was characterized by a lime mixture similar to stuccos and plasters. This CaO content was also the same in the level of RO_2_-(RO + R_2_O) correlation, which can be interpreted as an adjustment of the material differences based on CaO by the production times (Figure 10).

All the plaster samples had been illustrated in the domain close to the SiO_2_-CaO line, thereby displaying the tendency to be distributed in different locations depending on the SiO_2_ content (Figure 10A). The level of RO_2_-(RO + R_2_O) correlation also displayed a broader range of acidic oxides of the original plasters (Figure 10B), thereby implying that there was a slight difference in its composition.

### 4.2. Provenance Considerations

It is interpreted that all types of soils had been mixed for the calcareous materials since the Ca content is high and Si, Al, and K were detected except the original mortar. In particular, the original mortar was primarily produced using soil, while the new mortar was made by mixing some soil as the aggregate with the primary lime material.

**Figure 11:**
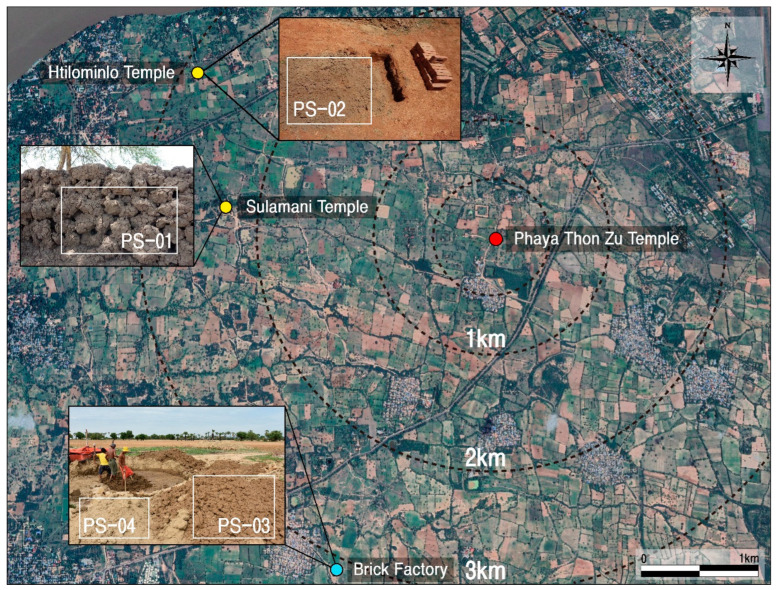
Locations showing the soil samples of origin for clay materials used to produce calcareous materials around the Phaya Thon Zu temple. Sample numbers are the same as those of Table 1.

Therefore, clayey and sandy soils were collected from the area around the Phaya Thon Zu temple to analyze their microstructure, mineral composition, and geochemical behavioral characteristics. This analysis enabled the examination of the clay used as a raw material for the calcareous materials, its origin, and the production technology system. Soil samples were collected from clay pits within a 3.5 km radius of the Phaya Thon Zu temple, which are currently used to produce bricks for various purposes, including repairing the temple site (Figure 11).

**Figure 12:**
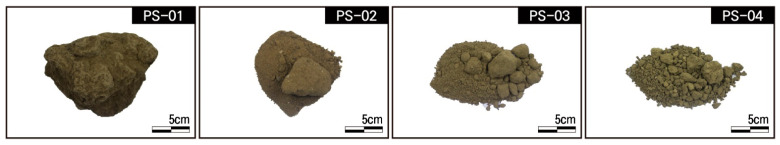
Occurrences of soil samples around the Phaya Thon Zu temple. Location of the collection point shown in Figure 11. Sample numbers are the same as those of Figure 11.

Sample PS-1 is viscous alluvium recovered from the marshy deposit in front of the Sulamani temple in its dried and peculiar state and produced in a lumpy form. PS-2 is a sandy subsoil distributed in the flat area in the vicinity of the Htillominlo temple (Figure 12). Samples PS-3 and PS-4 were collected during a visit to a brick factory in Thu Htay Kan village, situated approximately 3.5 km southwest of the Phaya Thon Zu temple.

These are the clay for the bricks that are used locally for the construction and repair of the temples as mentioned above (Figure 12). These marshy clays were collected at the suggestion of local experts. According to them, although there is no record in the literature, locals testified that in the past and present, the same materials were obtained from the same place in the same way and used to repair the monuments. Therefore, this study also respects their methods and reflects them in the study.

**Figure 13:**
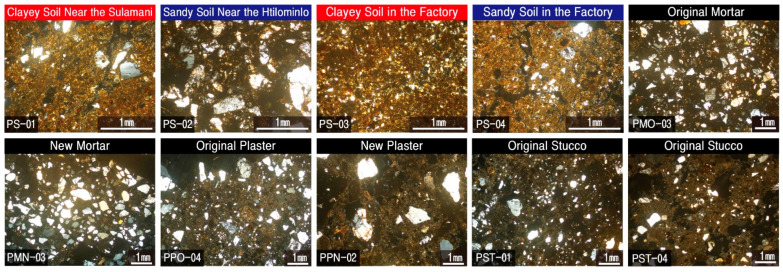
Microphotographs showing the polarizing microscopic images of soil and building materials around the Phaya Thon Zu temple. Sample numbers are the same as those of Figure 11.

In the Bagan region of Myanmar, tropical grassland (Savana) soil has developed due to the dry climate with low rainfall. Therefore, the soil layer is characterized by porosity and reddish color due to the large quantity of iron concentrated by leaching action due to rapid drainage. Accordingly, when the soil samples were observed under a polarizing microscope, they all displayed a reddish color and cryptocrystalline substrate, and quartz was regarded as the main mineral, while having slight differences in their particle sizes and contents (Figure 13).

Regardless of the production time, all of the studied mortars had relatively small particles of the constituent minerals compared to sandy soil. Still, the content of sand was observed to be high. While the stuccos and original plasters were characterized by a mineral distribution similar to that of clayey soil, the new plaster had characteristics that were most similar to those of sandy soil near Htillominlo temple, with mineral particle sizes of more than 1 mm (Figure 13).

**Figure 14:**
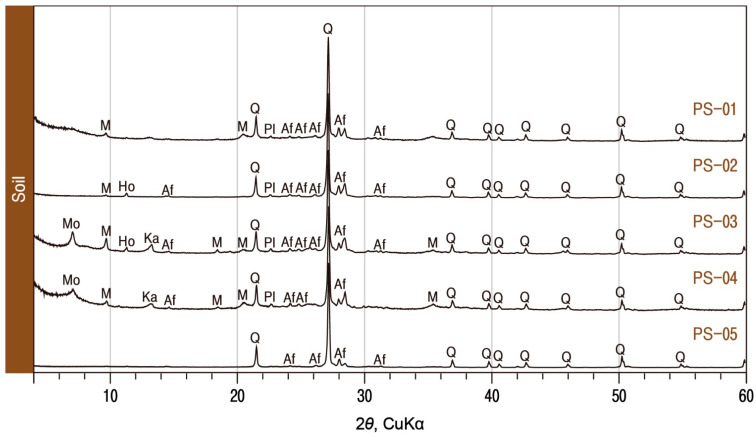
X-ray diffraction analysis of soil samples around the Phaya Thon Zu temple. Mo; montmorillonite, M; mica, Ka; kaolinite, Ho; hornblende, Af; alkali feldspar, Q; quartz, Pl; plagioclase. Sample numbers are the same as those of Figure 13.

When the homogeneity of these soil samples with calcareous materials was examined through X-ray diffraction analysis, it was found that they all contained quartz and alkali feldspar as major components, with the inclusion of trace amounts of mica and plagioclase (Figure 14). In addition, montmorillonite was detected in soils (PS-3 and PS-4) recovered from a brick factory in Thu Htay Kan. Thus, the calcareous materials used in the Phaya Thon Zu temple were found to have a very similar mineral composition to the soil except calcite. The results on calcareous substances obtained in the study are summarized in Table 5.

**Figure 15:**
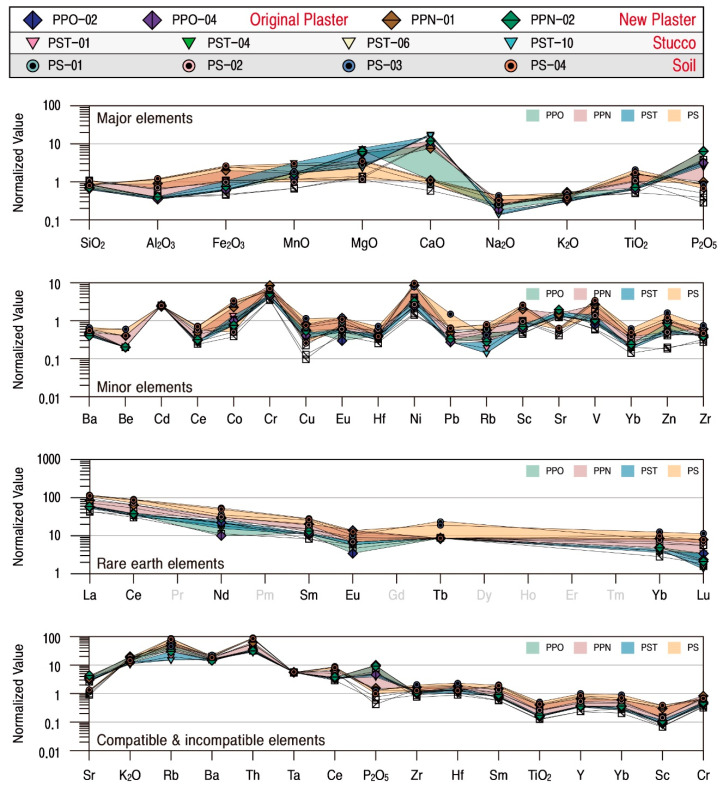
Normalized pattern diagrams showing the enrichment and deficiency factors of major, minor, rare earth, compatible and incompatible elements of calcareous materials around the Phaya Thon Zu temple. Sample numbers are the same as those of Table 1.

**Figure 16:**
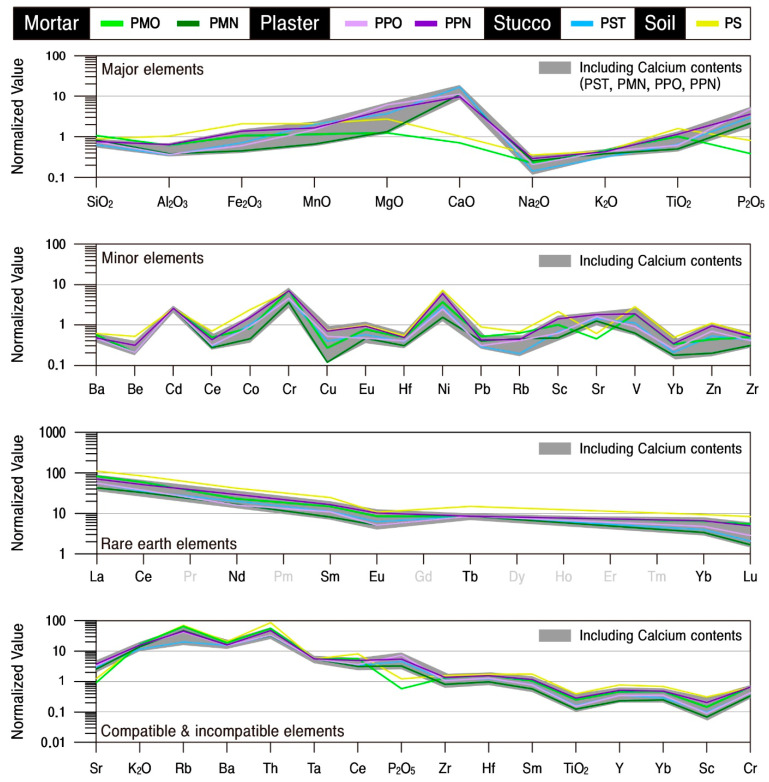
Normalized pattern diagrams showing the enrichment and deficiency factors of the major, trace, rare earth, compatible and incompatible elements by group of calcareous materials and soil around the Phaya Thon Zu temple. Sample numbers are the same as those of Table 1.

When the elemental behavior of the plasters and stuccos of the Phaya Thon Zu temple was compared with that of the soil, it was found that they had considerable geochemical homogeneity, thereby suggesting that they all utilized soil distributed in the general region of the temple (Figure 15). Therefore, the calcareous materials used in the temple were categorized according to the time of their production, and their behavior was examined by calculating the average values for the constituent elements of each material (Figure 16).

As a result, although the behaviors of rare earth elements were the same for all the groups, the main components, compatible and incompatible elements, displayed different levels of enrichment of CaO and P_2_O_5_ depending on whether lime was included or not. The new mortars, plasters, and stuccos containing lime were interpreted to have a higher CaO content due to the process of their production by mixing lime into the clay. At the same time, the P_2_O_5_ was believed to be a component generated as the result of organic matter being utilized as an additive in the process.

**Figure 17:**
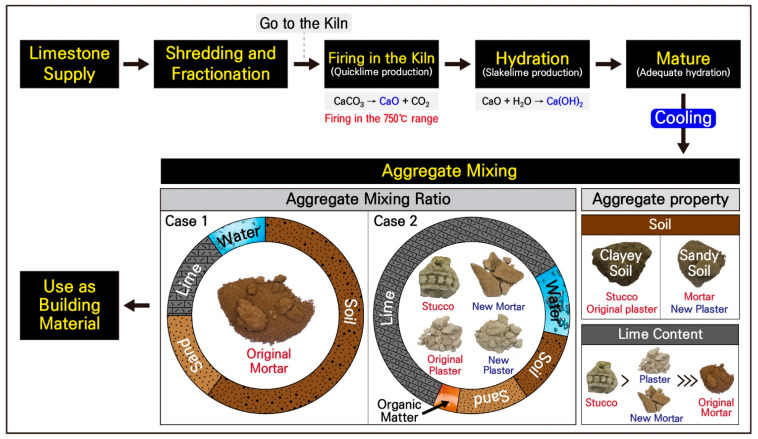
Schematic diagrams of the production technology system of calcareous materials using the construction of the Phaya Thon Zu temple.

The clay minerals detected in the soil mainly dissipated during the production process, and it appears that the lime additives were likely responsible for the detection of a high level of calcite in the new mortar, plaster, and stuccos (Figure 17). Accordingly, it can be concluded that the original materials utilized in the production of the calcareous materials were all soil from the temple sites that were homogeneous in their genesis, and it can be interpreted that the calcareous materials except for the original mortar were composed by adding lime to these clay materials.

### 4.3. Interpretation of Making Techniques

The calcareous materials exhibited the characteristics of lime mixtures except the original mortars, and thermal analysis confirmed a rapid weight loss in all of the materials at around 700 to 750 °C with an endothermic reaction due to the decarbonization of calcite. The calcite and quartz were mainly identified in the new mortars, plasters, and stuccos, and no detritus was observed in the milky white to white substrate based on their yield state and micro texture.

CaCO_3_ is the primary component of calcite that composes limestone, and calcite is the most stable form of calcium carbonate. Generally, when limestone is fired at high temperatures, it undergoes decarbonization and converts into quicklime (CaO), which is a steady condition. During this process, calcium carbonate, which is neutral and stable, escapes by decomposing into basic and unstable calcium oxide and acidic carbon dioxide.

Therefore, to obtain the lime needed for the new mortars, plasters, and stuccos, the firing of limestone must have taken place while refining the raw materials. In general, although calcite, a helpful mineral in limestone, begins to undergo phase decomposition at 650 °C, firing at 850 to 1000 °C is required to obtain higher-quality lime.

When limestone is heated at a temperature above 1000 °C, calcite produces quicklime (CaO) due to a calcination reaction involving the dissociation of carbon dioxide. Quicklime reacts with water to form slaked lime (Ca(OH)_2_), which in turn forms calcite (CaCO_3_) through a carbonation reaction in which carbon dioxide binds with slaked lime [15,16,17,18,21,29].

The utilization of lime as a construction material is based on the carbonization of slaked lime during this limestone cycle, and the calcite formed through this carbonization reaction acts as a mineral adhesive. Therefore, since it is advantageous to have a carbonization reaction to have high strength and durability, high-temperature firing is beneficial to obtain high-quality lime.

As a result of thermal analysis of the calcareous materials collected from the Phaya Thon Zu temple, a maximum endotherm was displayed in the temperature range of 710 to 730 °C due to the decarbonization of calcite, and no carbonation reaction with recrystallization was observed at temperatures higher than 900 °C. As such, it was found that pyrolysis ended at around 750 °C, and it was presumed that the calcareous material used in the temple was relatively low-quality lime that had undergone incomplete decarbonization in obtaining quicklime.

Moreover, the thermogravimetric analysis of each sample displayed a high weight loss of 20.95 wt.% for stuccos and 14.58 wt.% for plaster, and the average LOI was also increased to 20.37 wt.% for stuccos and 14.38 wt.% for plasters. This average LOI is very high compared to the average weight loss of 8.94 wt.% for the studied soil. Thus, although it can be interpreted that organic matter may have been used in the kneading of the lime and soil-based material mixture to produce the plasters and stuccos used in the temple, additional experiments are needed, along with additional review to confirm this.

By integrating the results of the material analysis of limestone and clay materials, which are the main raw materials utilized in the production of the studied calcareous materials, the production technology system was examined (Figure 17). First, the distribution would have been adjusted to make more straightforward utilization of the limestone quarried at the site of origin as construction material. The limestone was then transported to a nearby kiln, fired at a temperature below the range of 700 to 750 °C before undergoing a slaking process through a hydration reaction.

Although the hydration reaction is completed on the surface of the slaked lime particles, there may still be unreacted quicklime remaining inside the particles. Therefore, an additional aging process was possibly implemented to ensure sufficient hydration of all the particles. Aggregates are then mixed to function as a construction material, and the calcareous materials from the Phaya Thon Zu temple showed two different types of aggregate mixing ratios (Figure 17).

While the original mortars displayed characteristics close to normal soils, the other calcareous materials were produced by mixing lime-based materials with clay and organic materials. As a result of the previous comparison of the mineral composition of soils and each construction material, there were some differences in contents and particle sizes. However, the roundness is similar overall (Figure 13).

When the mixing ratio of the clay materials was examined through the results mentioned above, the minerals had relatively large particle sizes of more than 1 mm. In particular, it is likely that the sandy soil in the general region of the Htillominlo temple site was utilized for the production of the new plaster. However, since there is a slight difference in their contents, it is interpreted that some sorting process must have occurred.

Furthermore, since clayey soils appeared to have been used to produce the original plasters and stuccos, it was presumed that the mortar was produced by appropriately mixing clay and sandy soils. A more precise interpretation of the provenance of each material and the mixing ratio of clayey materials will require quantitative examination through experiments, along with the estimated provenance of lime materials.

## 5. Conclusions

1. The calcareous materials used in the construction of the Phaya Thon Zu temple in Bagan, Myanmar, are mortars, plasters, and stuccos, which were utilized as joint filler, plaster, and decorative elements inside and outside the temple. In the study, specimens of each calcareous material that fell off were collected by classifying their uses and production time, and their material characteristics and making techniques were discussed through mineralogical and geochemical analysis.
2. As a result of the integration of the material scientific characteristics of the studied calcareous specimens, the stuccos and plasters had lime-mixture characteristics due to their high content of CaO along with calcite. At the same time, the mortar was confirmed to have similar characteristics only in the new mortar used for repair. When their geochemical behavioral characteristics are examined, the clayey materials used in the construction were sourced from soils distributed in the temple site.
3. The calcareous auxiliary materials of the Phaya Thon Zu temple are a mixture of soil-based clay and lime paste, to which organic materials have been added. As a result of thermal analysis, in the firing of limestone performed during the process of obtaining the lime used to produce the calcareous materials, pyrolysis was found to have ended at around 750 °C. Therefore, relatively low-quality lime was obtained due to an incomplete decarbonization reaction while securing quicklime.
4. The aggregates mixed in producing the calcareous auxiliary materials were largely of two types. Although the original mortars generally had a composition close to the soil, other calcareous materials were produced with a higher lime content mixed with clay and organic matter. In particular, stuccos had the highest range of lime and organic matter among the calcareous materials and was combined with the most homogeneous selected aggregate. This reflects increased durability against weathering and ease of carving by considering its unique feature of being continuously exposed to the external environment.
5. If research on the clear provenance interpretation of the raw materials for calcareous auxiliary materials and investigation on the mixing ratio of clayey materials were to be carried out in the future based on the results of this study, it would be possible to establish conservational measures to maintain the outstanding universal value, authenticity, and integrity of the Phaya Thon Zu temple as a World Heritage Site.

## Figures and Tables

**Table 1:** Sample list of analytical materials and characteristics from the Phaya Thon Zu temple in Bagan historical area, Myanmar.

Type	Group	Sample No.	Location	Use
Mortars	PMO	PMO-1	On the outside surface of Phaya Thon Zu	Original mortars
		PMO-2		
		PMO-3		
	PMN	PMN-1		New mortars
		PMN-2		
		PMN-3		
Plasters	PPO	PPO-1	On the outside surface of Phaya Thon Zu	Original plasters
		PPO-2		
		PPO-3		
		PPO-4		
	PPN	PPN-1		New plasters
		PPN-2		
Stuccos	PST	PST-1	Near the Phaya Thon Zu temple	Original stuccos
		PST-2		
		PST-3		
		PST-4		
		PST-5		
		PST-6		
		PST-7		
		PST-8		
		PST-9		
		PST-10		
		PST-11		
Soils		PS-1	Near the Sulamani temple	Clayey soil
		PS-2	Near the Htilominlo temple	Sandy soil
		PS-3	Brick factory in the Thu Htay Kan	Clayey soil
		PS-4		Sandy soil

**Table 2:** Chemical compositions (wt.%) by SEM-EDS analysis of calcareous materials. Sample numbers are the same as those of Figure 6.

Sample	Si	Al	Fe	Ca	Mg	Na	K	Ti	O
Mortar 1	20.67	9.11	2.48	1.50	1.84	0.93	1.45	-	62.02
Plaster 2	16.57	2.48	-	16.42	3.52	-	1.39	-	0.43
Stucco 3	10.74	4.77	2.27	20.94	3.87	-	1.13	-	56.28

**Table 3:** Results of weight loss (wt.%) by thermal gravity analysis of calcareous materials. Sample numbers are the same as those of Figure 8.

Sample Name			Weight Loss	Sample Name			Weight Loss
Mortar	Original	PMO-2	3.31	Plaster	Original	PPO-2	12.28
		PMO-3	5.48			PPO-4	20.48
	New	PMN-1	14.02		New	PPN-1	8.27
		PMN-2	15.19			PPN-2	17.29
Stucco	Original	PST-1	21.52	Stucco	Original	PST-6	19.45
		PST-4	21.51			PST-10	21.33

**Table 4:** Chemical compositions of major elements (wt.%), some minor and rare earth elements (ppm) for calcareous materials. Sample numbers are the same as those of Table 1.

No.	Mortar				Plaster				Stucco			
	PMO-2	PMO-3	PMN-1	PMN-2	PPO-2	PPO-4	PPN-1	PPN-2	PST-1	PST-4	PST-6	PST-10
SiO_2_	78.66	76.64	60.63	59.42	61.62	48.58	59.27	51.78	43.98	44.83	49.17	45.87
Al_2_O_3_	8.72	8.60	5.47	5.24	5.4	4.88	12.33	5.54	5.64	4.71	5.01	4.71
Fe_2_O_3_	2.69	2.73	1.18	1.12	1.52	1.57	5.08	1.92	2.66	1.51	1.62	1.47
MnO	0.07	0.07	0.04	0.04	0.08	0.10	0.10	0.10	0.19	0.10	0.10	0.10
MgO	0.59	0.72	0.66	0.74	2.96	3.46	1.60	3.29	3.99	1.23	1.33	1.20
CaO	0.78	1.12	14.59	14.78	11.92	17.99	10.05	15.76	20.53	24.18	21.62	23.70
Na_2_O	0.71	0.70	0.80	0.79	0.73	0.57	1.02	0.79	0.54	0.41	0.43	0.41
K_2_O	2.58	2.46	2.08	2.12	2.97	1.82	2.01	2.72	1.59	1.74	1.94	1.67
TiO_2_	0.39	0.37	0.19	0.18	0.23	0.23	0.60	0.26	0.27	0.23	0.25	0.23
P_2_O_5_	0.05	0.09	0.07	0.71	1.18	0.58	0.18	1.13	0.59	0.46	0.63	0.52
LOI	3.26	5.35	14.60	14.98	12.11	20.14	8.13	17.14	20.66	21.31	18.67	20.84
Total	98.51	98.84	100.30	100.10	100.70	99.91	100.40	100.40	100.60	100.70	100.70	100.70
Ba	390	362	326	322	327	295	351	287	272	317	317	330
Be	1	1	1	1	1	1	2	1	1	1	1	1
Cd	0.5	0.5	0.5	0.5	0.5	0.5	0.5	0.5	0.5	0.5	0.5	0.5
Co	7	6	3	4	6	8	18	6	11	9	6	6
Cr	177	156	87	83	105	109	204	125	115	93	116	97
Cu	7	9	3	4	18	13	25	17	15	11	11	10
Hf	4	3.4	2.6	2.1	3.2	3	4.3	3.2	2.4	3.9	3.1	2.5
Ni	38	41	17	16	26	27	92	36	48	24	27	23
Pb	18	21	15	15	12	11	19	13	11	10	11	11
Rb	130	130	80	110	100	70	120	60	40	60	30	30
Sc	5.7	6.0	2.8	2.7	3.5	3.7	12.1	4.3	4.9	3.6	3.7	3.5
Sr	104	119	305	316	446	352	395	502	420	321	344	345
V	57	64	21	20	27	33	88	36	49	34	33	31
Zn	28	32	13	14	59	37	70	60	44	31	38	32
Zr	125	113	79	67	90	103	148	99	91	105	103	101
La	34.3	26.8	16.0	15.7	20.6	21.0	31.0	21.5	19.0	20.2	21.5	22.5
Ce	62	51	29	34	37	35	62	37	33	34	31	37
Nd	18	15	8	16.0	16	7	22	20	18	11	13	15
Sm	3.5	3.5	1.9	1.9	2.4	2.7	4.7	3.0	2.7	2.5	2.6	2.7
Eu	0.8	0.7	0.5	0.4	0.3	0.6	1.2	0.6	0.6	0.5	0.5	0.6
Tb	0.5	0.5	0.5	0.5	0.5	0.5	0.5	0.5	0.5	0.5	0.5	0.5
Yb	1.5	1.7	1.0	0.7	1.2	1.2	2.1	1.2	1.0	1.1	1.0	0.9
Lu	0.21	0.21	0.06	0.07	0.13	0.09	0.30	0.08	0.13	0.05	0.06	0.08

**Table 5:** Summary on X-ray diffraction analysis of calcareous and soil materials from the study area. Sample numbers are the same as those of Table 1.

Sample No.		Mo	M	Ho	Ka	Af	Q	He	Pl	Her	Ca
Soil	PS-1	-	*	-	-	**	***	-	*	-	-
	PS-2	-	*	*	-	**	***	-	*	-	-
	PS-3	*	*	*	*	**	***	-	*	-	-
	PS-4	*	*	-	*	**	***	-	*	-	-
Original Stucco	PST-1	-	*	*	-	**	***	-	*	-	**
	PST-4	-	-	-	-	**	***	-	-	-	**
	PST-6	-	*	-	-	**	***	-	-	-	**
	PST-10	-	-	-	-	*	***	-	-	-	**
Original Mortar	PMO-2	-	*	*	-	**	***	-	*	-	-
	PMO-3	-	*	*	-	**	***	-	*	-	-
New Mortar	PMN-1	-	*	-	-	**	***	-	*	-	**
	PMN-2	-	*	-	-	**	***	-	*	-	**
Original Plaster	PPO-2	-	-	-	-	**	***	-	*	-	**
	PPO-4	-	-	-	-	**	***	-	-	-	***
New Plaster	PPN-1	-	*	*	-	**	***	-	*	-	**
	PPN-2	-	-	-	-	**	***	-	*	-	**

Mo; montmorillonite, M; mica, Ho; hornblende, Ka; kaolinite, Af; alkali feldspar, Q; quartz, He; hematite, Pl; plagioclase, Her; hercynite, Ca; calcite. -: not detected, *: minor, **: moderate, ***: major.

## Data Availability

The original contributions presented in the study are included in the article, further inquiries can be directed to the corresponding author.
